# Immunomodulatory effect of NEDD8-activating enzyme inhibition in Multiple Myeloma: upregulation of NKG2D ligands and sensitization to Natural Killer cell recognition

**DOI:** 10.1038/s41419-021-04104-w

**Published:** 2021-09-04

**Authors:** Sara Petillo, Cristina Capuano, Rosa Molfetta, Cinzia Fionda, Abdelilah Mekhloufi, Chiara Pighi, Fabrizio Antonangeli, Alessandra Zingoni, Alessandra Soriani, Maria Teresa Petrucci, Ricciarda Galandrini, Rossella Paolini, Angela Santoni, Marco Cippitelli

**Affiliations:** 1 Department of Molecular Medicine, Sapienza University of Rome, Rome, Italy; 2 Department of Experimental Medicine, Sapienza University of Rome, Rome, Italy; 3 Wallace H. Coulter Department of Biomedical Engineering, Georgia Institute of Technology and Emory University, Atlanta, GA USA; 4 Institute of Molecular Biology and Pathology, National Research Council (CNR), Rome, Italy; 5 Hematology, Department of Clinical and Molecular Medicine, Sapienza University of Rome, Rome, Italy; 6 Istituto Pasteur-Fondazione Cenci Bolognetti, Rome, Italy; 7 IRCCS, Neuromed, Pozzilli, Italy

**Keywords:** Drug development, Myeloma, NK cells

## Abstract

Multiple Myeloma (MM) is an incurable hematologic malignancy of terminally differentiated plasma cells (PCs), where immune interactions play a key role in the control of cancer cell growth and survival. In particular, MM is characterized by a highly immunosuppressive bone marrow microenvironment where the anticancer/cytotoxic activity of Natural Killer (NK) cells is impaired. This study is focused on understanding whether modulation of neddylation can regulate NK cell-activating ligands expression and sensitize MM to NK cell killing. Neddylation is a post-translational modification that adds a ubiquitin-like protein, NEDD8, to selected substrate proteins, affecting their stability, conformation, subcellular localization, and function. We found that pharmacologic inhibition of neddylation using a small-molecule inhibitor, MLN4924/Pevonedistat, increases the expression of the NK cell-activating receptor NKG2D ligands MICA and MICB on the plasma membrane of different MM cell lines and patient-derived PCs, leading to enhanced NK cell degranulation. Mechanistically, MICA expression is upregulated at mRNA level, and this is the result of an increased promoter activity after the inhibition of IRF4 and IKZF3, two transcriptional repressors of this gene. Differently, MLN4924/Pevonedistat induced accumulation of MICB on the plasma membrane with no change of its mRNA levels, indicating a post-translational regulatory mechanism. Moreover, inhibition of neddylation can cooperate with immunomodulatory drugs (IMiDs) in upregulating MICA surface levels in MM cells due to increased expression of CRBN, the cellular target of these drugs. In summary, MLN4924/Pevonedistat sensitizes MM to NK cell recognition, adding novel information on the anticancer activity of neddylation inhibition.

## Introduction

Neddylation is a post-translational modification that adds a ubiquitin-like protein, NEDD8 (neuronal precursor cell-expressed developmentally downregulated protein 8), to selected substrate proteins, affecting their stability, conformation/function, and subcellular localization; this in turn can regulate many biological and pathological processes, including cancer progression and immune response [1–4].

This pathway involves the activity of NAE (NEDD8-activating enzyme, NAE1-UBA3), an enzyme required for the adenylation and activation of the ubiquitin-like molecule NEDD8 [5–7], which is then transferred to a lysine residue of the target protein by the coordinate action of an E2 NEDD8-conjugating enzyme (UBE2M or UBE2F) [8, 9] and in the final step, NEDD8-E3 ligases catalyze the transfer of NEDD8 from E2 to the target protein [10]. Most reported NEDD8-E3 ligases contain the RING domain, among which the best studied are RBX1/ROC1 and its homologue RBX2/SAG [11]. In this context, neddylation is required for the activation of the Cullin Ring Ligases (CRLs), the largest family of E3-ubiquitin ligases whose regulated activity can control the degradation of about 20% of proteasome-regulated proteins [11–14]. CRLs are multiprotein complexes composed by a Cullin subunit, to which NEDD8 is covalently attached, which act as a molecular scaffold able to bind to an adaptor protein and a substrate receptor at its N-terminus, and a RING protein at its C-terminus. Overactivation of CRLs has been well described in cancer and can lead to tumor growth and progression. Moreover, the enzymes implicated in the neddylation pathway (e.g., NAE1/UBA3, UBE2M/UBE2F, NEDD8-E3 ligases) are often overexpressed in different human cancers. This correlates with disease progression and poor patient survival [15–21]; thus, targeting Cullin neddylation represents an attractive approach for cancer treatment [2, 22, 23].

Multiple Myeloma (MM) is an incurable hematologic malignancy of terminally differentiated plasma cells, where immune interactions play a key role in the control of MM growth and survival. In this regard, MM is characterized by a highly immunosuppressive bone marrow (BM) microenvironment where Natural Killers (NK) cells functionality is impaired [24, 25]. NK cells are innate lymphoid cells provided of a repertoire of germline-encoded activating and inhibitory receptors thanks to which they can distinguish healthy cells, from the infected/stressed or transformed ones. After activation, they have the ability to recognize and kill cancer cells and to produce many different cytokines and chemokines [26, 27]. Immunosurveillance against MM onset and progression is actively mediated by NK cells, and among the activating receptors responsible for NK cell recognition and killing of MM cells, the most relevant are NKG2D, DNAM-1, and the NCRs (NKp46, NKp30, NKp44) [28–30]. On the other hand, MM can directly inhibit NK cell functions by producing immune suppressive factors and/or reducing their susceptibility to NK cell recognition. In addition, MM cells can undergo decreased surface expression of NK cell-activating ligands (e.g., NKG2DLs) [31, 32], while expressing (together with other cell populations in the bone marrow) ligands of inhibitory receptors, such as the ligand of PD-1 (PD-L1) [33, 34], providing a mechanism of tumor escape. Thus, improving NK cell responsiveness may be a promising therapeutic approach to treat MM; in particular, the modulation of the balance between the activating and inhibitory NK cell signals and the sensitization of cancer cells to NK cell-mediated recognition and cytotoxicity may significantly contribute to enhance anti-myeloma immune responses.

In this work, we describe inhibition of neddylation as a regulator of the expression of the NKG2DL MICA and MICB in MM cells, making these cells more efficient to activate NK cell degranulation.

Mechanistically, we found that inhibition of neddylation upregulates cell surface expression of these ligands at different levels, transcriptional for MICA and post-translational for MICB. In addition, inhibition of neddylation can cooperate with immunomodulatory drugs (IMiDs) in the upregulation of MICA expression, further extending their possible therapeutic combinations.

In conclusion, these findings provide new insights on the immuno-mediated antitumor activities of neddylation inhibition in MM and further elucidate the molecular mechanisms that regulate NK cell-activating ligand expression in MM.

## Results

### Inhibition of neddylation upregulates NKG2DLs MICA and MICB expression on human MM cells and enhances their recognition by NK cells

**Fig. 1:**
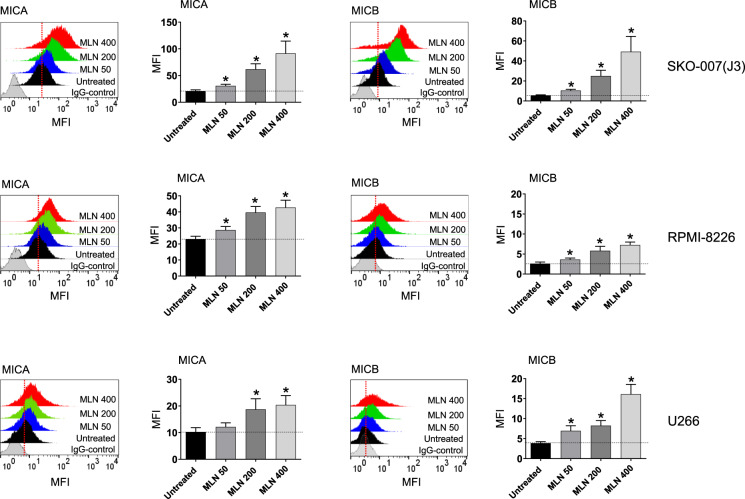
MLN4924 upregulates MICA and MICB expression in human MM cell lines. MICA and MICB cell surface expression was analyzed by flow cytometry on SKO-007(J3), RPMI-8226 and U266 cells treated with MLN4924 (50–400 nM) for 72 h. Representative overlays are shown. Histograms represent the average of the mean fluorescence intensity (MFI) values of the indicated ligand (with treatment-specific isotype control MFI subtracted out). The MFI of MICA and MICB were calculated based on at least four independent experiments and statistical significance (±SEM) was evaluated by paired Student *t* test (*P < 0.05).

**Fig. 2:**
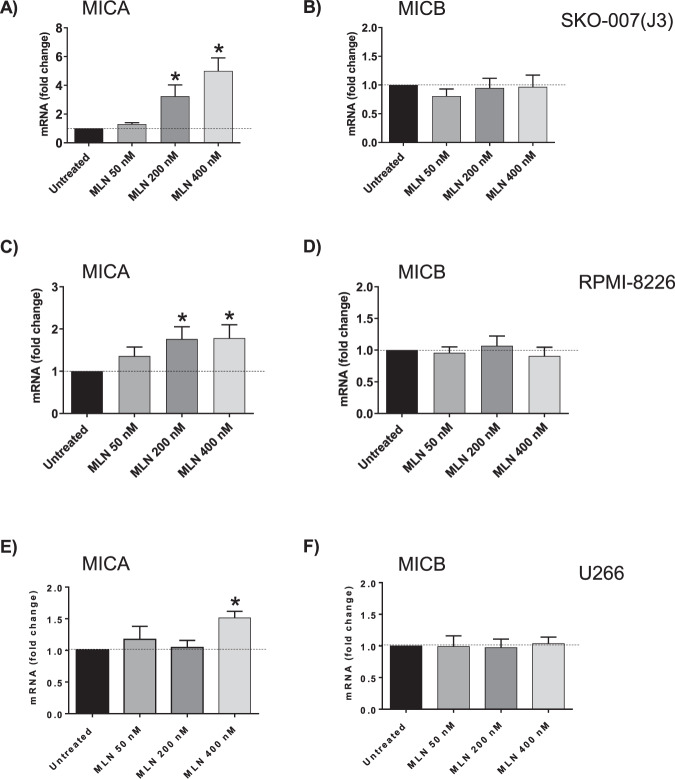
MLN4924 upregulates MICA but not MICB mRNA levels in human MM cell lines. Real-time PCR analysis of total mRNA extracted from SKO-007(J3), RPMI-8226 and U266 cells, untreated or treated with the indicated concentrations of MLN4924 as described above, for 48 h. (A,C,E) MICA. (B,D,F) MICB. Data, expressed as fold change units, were normalized to GAPDH, and were referred to the untreated sample considered as calibrator (mean ± SEM of four independent experiments *P < 0.05).

**Fig. 3:**
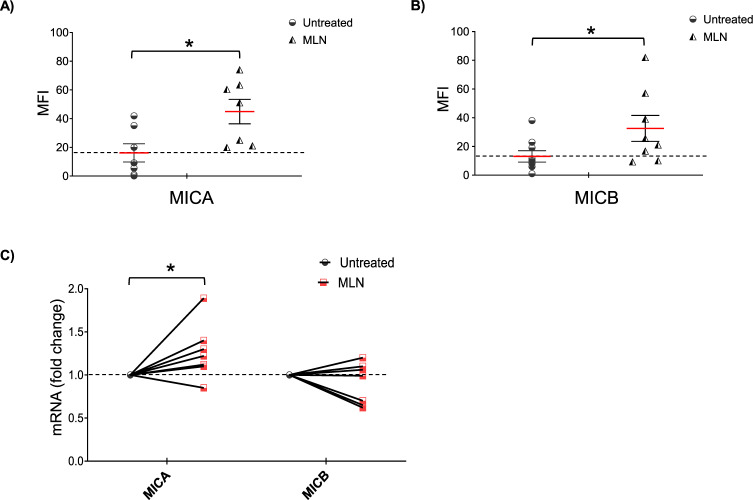
Upregulation of MICA and MICB expression on patient-derived PCs upon MLN4924 treatment. **A**, **B** MICA and MICB cell surface expression was analyzed by flow cytometry on patient-derived MM cells treated with 400 nM MLN4924 for 72 h. Histograms represent the average of the mean fluorescence intensity (MFI) values of the indicated ligand (with treatment-specific isotype control MFI subtracted out). The MFI of MICA and MICB were calculated based on at least four independent experiments and statistical significance was evaluated (±SEM) by paired Student *t* test (*P < 0.05). **C**) Real-time PCR analysis of total mRNA extracted from purified CD138^+ ^cells untreated or treated with MLN4924 as described above for 48 h in complete medium supplemented with 20 ng/ml IL-3 and 2 ng/ml IL-6. Data, expressed as fold change units, were normalized to GAPDH and were referred to the untreated sample considered as calibrator (*P < 0.05). Myeloma cells were selected using anti-CD138 magnetic beads and more than 95% of the purified cells expressed CD138 and CD38.

We investigated the role mediated by neddylation on the expression of NK cell-activating ligands in MM. To this purpose, a panel of human MM cell lines already shown to express NKG2DLs [SKO-007(J3), RPMI-8226, U266, ARP-1, JJN-3] [35, 36] were treated for 72 h with MLN4924/Pevonedistat (nanomolar range), a small-molecule inhibitor of NEDD8-activating enzyme (NAE) currently in phase I/II/III clinical trials for patients suffering from solid and haematological malignancies, including MM [37–42]. MLN4924 is structurally related to adenosine monophosphate (AMP) and after the binding to the NAE active site, it forms a covalent adduct (NEDD8-MLN4924) which resembles NEDD8-adenylate and blocks NAE activity. FACS analysis shown in Fig. 1 (and Suppl. Figure 1), indicates that MLN4924 upregulates cell surface expression of the NKG2D ligands MICA and MICB on MM cell lines expressing different basal levels of these proteins. Surprisingly, analyzing the cell lines showing the higher induction of these ligands, we noticed a significant increase of MICA mRNA levels after 48 h treatment with MLN4924, with no relevant effect on MICB mRNA levels (Fig. 2) indicating different regulation of these two ligands by neddylation. Concerning the other NKG2D ligands (ULBPs), the cell lines tested express low ULBP1 levels, low/undetectable levels of ULBP2/5/6 and ULBP3, and the treatment with MLN4924 did not significantly modify their cell surface levels (Supplementary Fig. 2). MLN4924 treatment at the concentration used in the majority of our experiments (200 nM), only minimally affected cell viability of these cell lines after 72 h treatment, as assessed by Annexin-V staining (Supplementary Fig. 3). In addition, we confirmed these results also in CD138^+ ^MM cells isolated from the bone marrow of MM patients (Table 1), showing higher cell surface expression of MICA and MICB following treatment with MLN4924 (Fig. 3A and B and Supplementary Fig. 4). Accordingly, also in this case MLN4924 upregulated MICA mRNA levels, while MICB mRNA levels were not affected (Fig. 3C).

**Table 1:** Clinical parameters of MM patients.

Patient no.	Sex/Age	Clinical stage	Monoclonal Ig	% PCs in BM
1	M/62	Onset	IgG-k	42%
2	F/59	Onset	IgA-k	51%
3	M/68	Relapse	IgG-λ	31%
4	F/74	Relapse	IgG-λ	47%
5	M/56	Onset	IgG-k	8%
6	F/83	Relapse	IgG-k	38%
7	F/79	Relapse	IgG-λ	46%
8	M/89	Onset	IgG-k	50%
9	F/61	Onset	IgG-k	37%
10	F/72	Onset	IgG-k	48%
11	F/78	Progression	IgG-k	8%
12	M/71	Relapse	IgG-k	61%
13	F/76	Onset	IgG-λ	62%
14	F/82	SMM	IgA-λ	19%
15	F/70	Onset	micro-k	38%
16	F/53	Onset	IgG-λ	32%
17	F/82	Onset	IgA-λ	35%
18	F/82	Relapse	IgG-k	23%
19	F/70	Onset	micro-λ	24%
20	F/65	Relapse	IgG-k	65%

Patients were classified according to the status of disease. The percentage of plasma cells in the BM and monoclonal Ig is indicated.

**Fig. 4:**
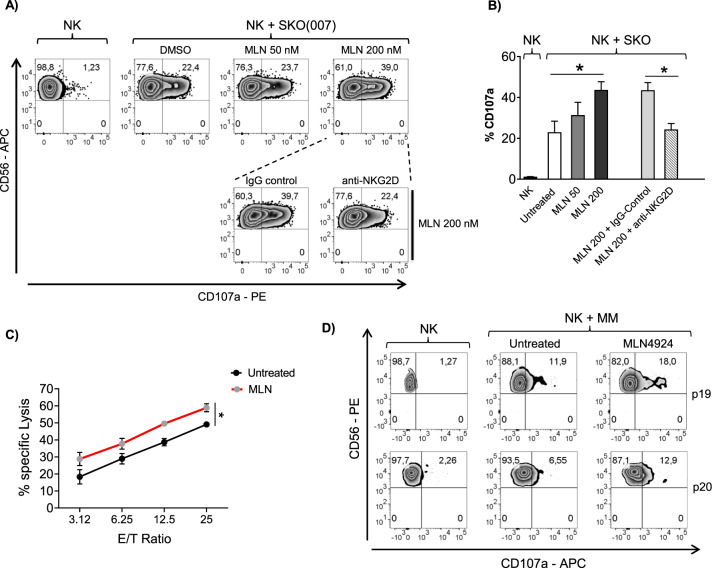
Increased susceptibility of MLN4924-treated MM cells to NK cell recognition and degranulation. Primary cultured NK cells obtained from PBMCs isolated from healthy donors, were incubated with SKO-007(J3) cells, untreated or treated with the indicated concentration of MLN4924 for 72 h as described above and used as target cells in a degranulation assay. The assay was performed at the Effector:Target (E:T) ratio of 2.5:1. After 2 h at 37 °C, cells were stained with anti-CD56, anti-CD3 and anti-CD107a mAbs. Cell surface expression of CD107a was analyzed on FSC/SSC-gated and CD56 ^+^ CD3^−^ cells. To evaluate the role of NKG2D, cells stimulated with the highest dose of MLN4924, were incubated with NK cells pretreated with anti-NKG2D blocking antibody or IgG control. **A** Representative degranulation. **B** Percentage of CD107a positive cells was calculated based on four independent experiments and statistical significance was evaluated by paired Student *t* test (*P < 0.05). **C** Primary cultured NK cells from different donors were incubated with SKO-007(J3) cells untreated or treated with MLN4924 (200 nM) for 48 h and used as target cells in 7-AAD/CFSE cell-mediated cytotoxicity assays. These assays were performed in triplicate at the Effector:Target (E:T) ratios ranging from 3.125 to 25:1. **D**) CD138- bone marrow cells, cultured for 2 days in complete medium supplemented with IL-2 (200 U/mL), were incubated with purified autologous myeloma cells, untreated or treated with MLN4924 as described above, and used as target cells in a degranulation assay. The assay was performed at the Effector:Target (E:T) ratio of 2.5:1. After 2 h at 37 °C, cell surface expression of CD107a was analyzed on CD56^+ ^CD16^+ ^CD3^−^ cells. Results obtained from two patients (P19 and P20) are represented.

To investigate the functional consequences of MLN4924-induced changes of MICA/B expression, we analyzed by flow cytometry the cell surface upregulation of the lysosomal marker CD107a (a surrogate marker for cytotoxic granule exocytosis) on primary cultured NK cells, isolated from different healthy donors, upon interaction with SKO-007(J3) cells, untreated or treated with the drug. As shown in Fig. 4A and B, basal expression of CD107a on NK cells was increased when co-cultured with SKO-007(J3) target cells previously exposed to MLN4924; this effect was significantly inhibited by a blocking anti-NKG2D mAb, indicating that induction of NK cell degranulation is NKG2D-dependent. In addition, these observations were also confirmed using 7-AAD/CFSE cell-mediated cytotoxicity assays, where SKO-007(J3) treated cells were lysed more efficiently by NK cells compared to untreated controls (Fig. 4C). Accordingly, a higher capability of degranulation was also observed in patient-derived NK cells against MLN4924-treated autologous MM target cells (Fig. 4D). Altogether, these data indicate that inhibition of neddylation enhances MICA/B cell surface expression in MM cells, increasing their susceptibility to NK cell recognition, degranulation and killing.

### Molecular mechanisms underpinning MLN4924-induced upregulation of MICA/B in MM cells

**Fig. 5:**
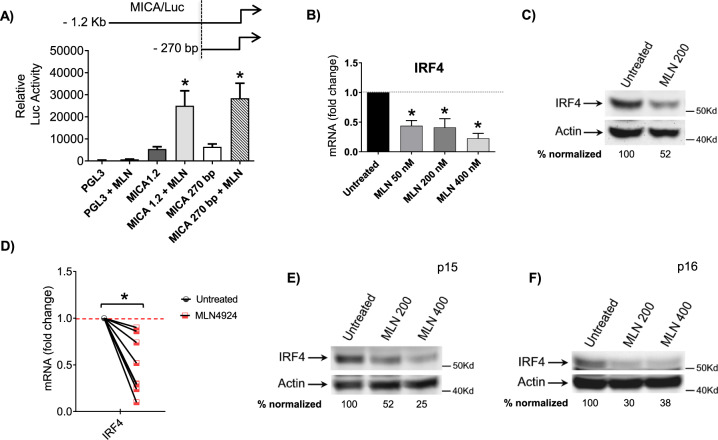
The MICA gene promoter and the transcriptional repressor IRF4 are targets of NAE inhibition. **A** SKO-007(J3) cells were cotransfected with 5 µg of the indicated luciferase reporter vector and 1 µg of pRL-TK. After electroporation cells were treated with MLN4924 (200 nM) for 48 h, harvested and protein extracts were prepared for the Luciferase and Renilla assays. Results are expressed as Relative Luciferase activity normalized to protein concentration as well as to Renilla activity produced off the internal control plasmid and represent the mean value (±SEM) of four independent experiments (*P < 0.05). A schematic representation of the progressive MICA promoter deletions used in these experiments is shown. **B** Real-time PCR analysis of total mRNA obtained from SKO-007(J3) cells, untreated or treated with the indicated concentrations of MLN4924 for 48 h. Data, expressed as fold change units, were normalized to GAPDH, and were referred to the untreated sample considered as calibrator, represent the mean (±SEM) of four experiments (*P < 0.05). **C** Western-blot analysis of IRF4 in SKO-007(J3) cells untreated or treated with MLN4924 for 48 h. β-Actin was used as protein-loading control. One representative western-blot out of three independent experiments is shown. Densitometric analysis of the reported Western blot is shown. **D** Real-time PCR analysis of total mRNA obtained from purified CD138^+ ^cells untreated or treated with MLN4924 (400 nM) as described above for 48 h in complete medium supplemented with 20 ng/ml IL-3 and 2 ng/ml IL-6. Data expressed as fold change units, were normalized to GAPDH, and referred to the untreated sample considered as calibrator. **E**, **F** Lysates of MM cells purified from two patients (CD138^+^ cells) untreated or treated with MLN4924 (200 and 400 nM) for 48 h in complete medium supplemented with 20 ng/ml IL-3 and 2 ng/ml IL-6, were subjected to Western Blotting using anti-IRF4 and anti-βActin antibodies. Densitometric analysis of normalized IRF4/Actin is shown.

**Fig. 6:**
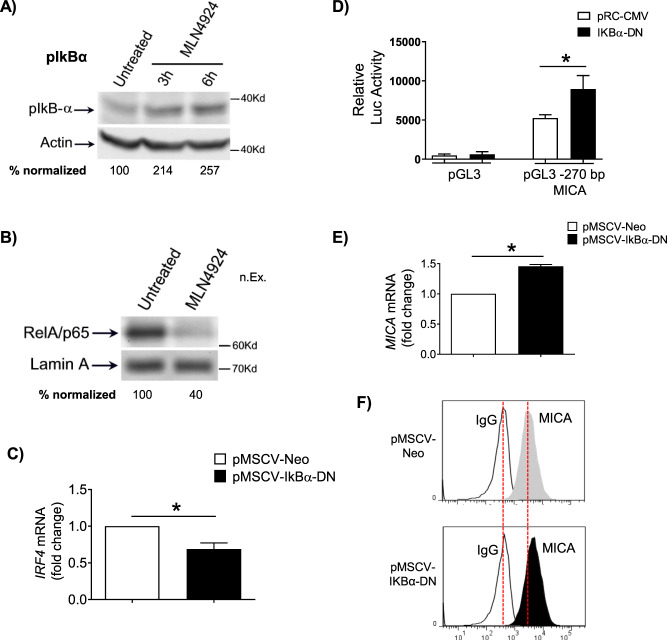
Role for neddylation in the regulation of the NF-kB/IRF4/MICA axis. **A** MLN4924 (200 nM) stabilized the expression of pIkBα and **B**) repressed nuclear translocation of p65/RelA in SKO-007(J3) cells treated for 48 h. Data are representative of one out of two independent experiments. Densitometric analyses of normalized pIkBα or p65/Actin are shown. **C** IRF4 mRNA levels were quantified by Real-time PCR in SKO-007(J3) cells overexpressing a dominant-negative form of IkBα (IkBα-DN) or the empty control vector pMSCV-Neo. Data, expressed as fold change units, were normalized to GAPDH, and referred to the pMSCV-Neo infected cells considered as calibrator and represent the mean (± SEM) of 3 experiments (*P < 0.05). **D** SKO-007(J3) cells were transiently cotransfected with the indicated MICA promoter deletion and an expression vector for IkBα-DN (S32A/S36A) or the empty control vector (pRc-CMV). Results are expressed as Relative Luciferase activity normalized to protein concentration as well as to Renilla activity produced off the internal control plasmid and represent the mean value (±SEM) of three independent experiments (*P < 0.05). **E**–**F** MICA mRNA and cell surface expression were analyzed in SKO-007(J3) cells overexpressing a dominant-negative form of IkBα (IkBα-DN) as described above. Data shown in (**E**), expressed as fold change units, were normalized to GAPDH and referred to the pMSCV-Neo infected cells considered as calibrator, represent the mean (±SEM) of 3 experiments (*P < 0.05). A representative FACS analysis of MICA expression in infected cells is shown in (**F**).

**Fig. 7:**
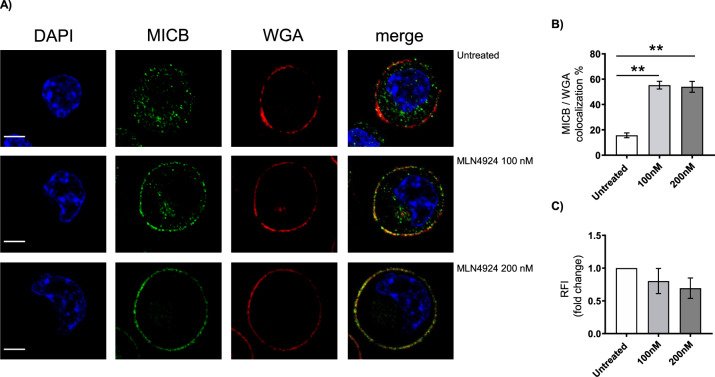
Cellular relocalization of MICB by MLN4924 in MM cells. **A** MICB expression analyzed by confocal microscopy in SKO-007(J3) cells untreated or treated with the indicated concentrations of MLN4924 for 72 h. Cells were fixed with paraformaldehyde and plasma membrane (in red) was stained with Alexa Fluor 594-conjugated Wheat Germ Agglutinin (WGA). After permeabilization cells were stained with anti-MICB followed by Alexa Fluor 488-conjugated goat antimouse IgG (in green). Nuclei were stained with DAPI (in blue). A representative image of SKO-007(J3) cells treated with MLN4924 or vehicle is reported is shown. The overlay of the two-color merged image (yellow/red) is shown. Scale bar 5 μm. **B** Percentage of colocalization of MICB with WGA. Histograms represent the means (±SD) of colocalization indexes analyzed on randomly acquired fields from three independent experiments (**P < 0.001). **C** Relative Fluorescence Intensity (RFI) (whole cell) analyzed on randomly acquired fields of 3 independent experiments. Histograms indicate the mean ± SD.

To explore the possibility that neddylation inhibition could increase MICA expression at the transcriptional level, we analyze the effects of MLN4924 on SKO-007(J3) cells transiently transfected with different MICA 5’-flank promoter-fragments, cloned upstream of a luciferase reporter gene. As shown in Fig. 5A, the drug enhanced the activity of the progressive promoter deletions (−1.2Kb to −270bp from the translation start site), delimiting a minimal fragment spanning from −270bp still responsive to MLN4924. In this regard, our previous observations identified IRF4, IKZF1, and IKZF3 as IMiDs “druggable” transcriptional repressors of NK cell-activating ligands in MM cells [35], and they have been proven to inhibit the activity of the −270bp/MICA promoter deletion [35]. We investigated whether MLN4924 could modulate the expression of these transcription factors (TFs) in our experimental setting, and we found IRF4 to be significantly downregulated at mRNA and protein level after neddylation inhibition (Fig. 5B and C). These data were also confirmed in different MM patient-derived malignant plasma cells (CD38^+ ^CD138^+^) both for mRNA and protein expression (Fig. 5D–F). Modulation of IRF4 by MLN4924 has been described in different models [38], as the result of the inhibition of CRL1^βTRCP^ activity. Indeed CRL1^βTRCP^ regulates NF-κB activation through the ubiquitination and degradation of its inhibitor IκBα, and IRF4 is a direct transcriptional target of NF-κB [43–45]. As shown in Fig. 6A and B, downregulation of IRF4 by MLN4924 correlated with increased levels of pIkBα and lower nuclear translocation of the NF-κB subunit RelA/p65 in SKO-007(J3) cells. Accordingly, overexpression of a IkBα dominant negative (S [32A]/S [36A]) resulted in lower IRF4 mRNA levels (Fig. 6C) and upregulated promoter activity, mRNA levels and cell surface expression of MICA (Fig. 6D–F), configuring MLN4924 as a modulator of the NF-kB/IRF4/MICA axis. Moreover, we also analyzed the expression of IKZF3 and IKZF1, two TFs required for MM growth and survival [46], which are known to negatively control the functional properties of many immune cells [46], and to repress MICA gene expression in MM [35, 47]. As shown in Supplementary Fig. 5A and B, MLN4924 induced a significant inhibition of IKZF3 in SKO-007(J3) cells; interestingly no relevant effects were observed for IKZF1 in the same experimental setting (Suppl. Figure 5C and D). These data were also confirmed in different MM patient-derived malignant plasma cells (CD38^+ ^CD138^+^) both for mRNA and protein expression (Suppl. Figure 5E–G). Concerning the effect of neddylation on MICB cell surface expression in the absence of relevant changes of mRNA expression, NKG2DL on tumor cells can be regulated by different post-translational mechanisms [48]. Indeed, active intracellular trafficking has been shown to regulate the expression of MICB [49]. We asked if the inhibition of neddylation by MLN4924 could affect MICB cellular distribution in MM cells. We treated SKO-007(J3) cells with MLN4924 for 72 h and analyzed its cellular localization through confocal microscopy. Interestingly, as shown in Fig. 7A and B, we found a significant accumulation of MICB to the cell surface as revealed by the colocalization with WGA, a carbohydrate-binding lectin that has a high affinity for sialic acid and glycoproteins on cell surface membranes [50]. This was not related to increased total ligand levels in treated cells (Fig. 7C), confirming the involvement of post-translational mechanisms in the upregulation of MICB membrane expression.

The overall data indicate that MICA promoter activity, mRNA and cell surface expression are enhanced by inhibition of neddylation and that MICB accumulates on the cell surface with no changes of its mRNA or cellular protein expression, highlighting a different regulation of these proteins by neddylation.

### Narrowing selectivity: DCN1 inhibition recapitulates the upregulation of MICA/B by MLN4924 in MM cells

CRLs regulate homeostasis of ~20% of cellular proteins and their activation requires neddylation of their respective cullin subunit; in this regard, selective targeting of neddylation of individual cullin members could represent an attractive strategy for the specific control of certain cellular proteins. Recent reports have described the development of novel small molecules endowed with selective pharmacologic modulation of the N-terminal acetylation-dependent regulatory interaction of UBE2M with DCN1 (DCUN1D1/SCCRO) [51, 52], a regulatory subunit of the multiprotein E3 ligase for the ubiquitin-like protein NEDD8. This allows a more targeted and nuanced activity relative to the complete ablation of neddylation afforded by MLN4924 or other NEDD8 E1 inhibitors, with preferential selectivity toward CUL1 and CUL3 [52, 53]. Based on these premises, we investigated the activity of the pharmacologic inhibitor of DCN1-UBE2M complex, NAcM-OPT, a small molecule with high level of selectivity for inhibition of DCN1 over the highly homologous human DCN isoforms [52, 53]. As shown in Supplementary Figure 6A,B and F,G, treatment of SKO-007(J3) cells with NAcM-OPT (micromolar concentration range, previously shown to be effective in cell-based studies [52]), significantly increased cell surface expression of MICA and MICB, as detected by flow cytometry. In this experimental setting, the effect on MICA upregulation on the plasma membrane correlated with increased levels of MICA mRNA (Suppl. Figure 6C), and, similarly to MLN4924-mediated NAE inhibition, with decreased levels of the MICA repressors IRF4 and IKZF3 (Suppl. Figure 6D and E), although to a lower level than that observed using MLN4924. In agreement with the data obtained using MLN4924, no differences were observed for MICB mRNA expression (Suppl. Figure 6H). These data indicate that selective inhibition of CRLs can regulate NKG2DL expression in MM cells.

### Neddylation inhibition enhances IMiDs immunomodulatory activity in MM cells

**Fig. 8:**
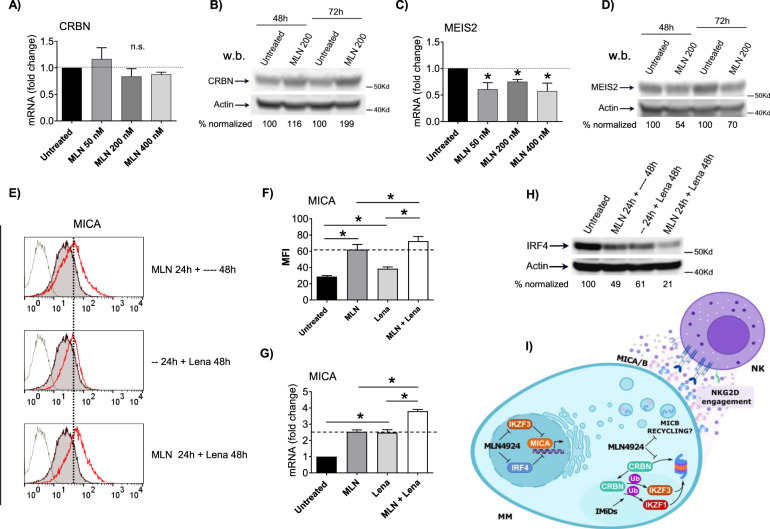
Neddylation inhibition increases upregulation of MICA induced by IMiDs. **A**, **C** Real-time PCR analysis of total mRNA obtained from SKO-007(J3) cells, untreated or treated with the indicated concentrations of MLN4924 for 48 h. Data, expressed as fold change units, were normalized to GAPDH, and referred to the untreated cells considered as calibrator, represent the mean ± SEM of 3 independent experiments (*P < 0.05). **B**, **D** Western blot analysis of CRBN (**B**) and MEIS2 (**D**) in SKO-007(J3) cells untreated or treated with MLN4924 for 48/72 h. β-Actin was used as protein-loading control. Data are representative of one out of two independent experiments. Densitometric analyses of the reported Western blots are shown. **E** MICA cell surface expression was analyzed by flow cytometry on SKO-007(J3) cells treated with MLN4924 (200 nM) and/or Lenalidomide (5 µM) following the indicated scheme. (–) and (—) indicate respectively the treatment with vehicle for 24 h and 48 h. After the first 24 h of MLN4924 or vehicle treatment, cells were washed twice in complete medium, reseeded, and stimulated with Lenalidomide or vehicle for additional 48 h. Representative overlays are shown. **F** Histograms represent the average of the mean fluorescence intensity (MFI) values of the indicated ligand (with treatment-specific isotype control MFI subtracted out). The MFI of MICA is representative of at least four independent experiments as shown in (**E**) and statistical significance was evaluated by paired Student *t* test (*P < 0.05). **G** Real-time PCR analysis of total mRNA obtained from SKO-007(J3) cells, untreated or treated as described above with MLN4924 (200 nM) and/or Lenalidomide (5 µM). Data, expressed as fold change units, were normalized to GAPDH, and referred to the untreated sample considered as calibrator, represent the mean (±SEM) of 3 experiments (*P < 0.05). **H** Western blot analysis of IRF4 in SKO-007(J3) cells untreated or treated with MLN4924 (200 nM) for 24 h and/or Lenalidomide (5 µM) for 48 h. β-Actin was used as protein-loading control. Data are representative of one out of two independent experiments. Densitometric analysis of the reported Western blot is shown. **I** Model: inhibition of neddylation and regulation of MICA/B expression on MM cells. The present study provides evidence that inhibition of neddylation increases NKG2D ligand MICA and MICB expression in MM via transcriptional and post-translational mechanisms respectively and suggests possible cooperation with IMiDs in this pathway.

Previous work from other laboratories has shown that CRBN can be targeted and degraded by CUL1/SCF^Fbxo7^ E3-ubiquitin ligase and proteasome [54]. In this context, pretreatment with either NAE inhibitors or proteasome inhibitors (e.g., Bortezomib) has been shown to increase CRBN cellular levels because it can no longer be degraded. This leads to an enhanced sensitivity to IMiDs and MM cytotoxicity in preclinical studies and improves clinical responses [55]. We hypothesized that a similar drug treatment scheme could reproduce the observed effects from an immunomodulatory point of view, and further increase the IMiDs activity toward NKG2DL expression in MM cells [35]. To address this point, we analyzed the expression levels of CRBN in SKO-007(J3) cells in the presence of increasing concentrations of MLN4924. Data shown in Fig. 8A, indicate that treatment with MLN4924 did not change CRBN mRNA levels, but significantly increased its protein expression (Fig. 8B). In the same experimental setting, MLN4924 downregulated both mRNA and protein levels of MEIS2 (Fig. 8C and D), a transcription factor previously identified as an endogenous cellular substrate of the E3-ubiquitin ligase complex CRL4-cereblon (CRL4^CRBN^) [56]. Importantly, MEIS2 has been described by our group as a regulator of proliferation, cell death, and activity of IMiDs in MM, given its ability to regulate the expression of IRF4 and IKZF3, and to act as an endogenous competitor for CRL4^CRBN^ [47].

We focused our attention on MICA expression (MICB expression is not affected by Lenalidomide in MM cells [35]) and, as shown in Fig. 8E–G, pretreatment with MLN4924 for 24 h followed by stimulation with Lenalidomide significantly potentiated MICA cell surface and mRNA expression, confirming our hypothesis. Accordingly, the combination of MLN4924 plus Lenalidomide further downregulated cellular levels of the MICA repressor IRF4 (Fig. 8H). Overall, these data further extend the preclinical and clinical observations of enhanced sensitivity to IMiDs after treatment with neddylation or proteasome inhibitors in MM

## Discussion

Neddylation inhibition has been shown to exert antitumor activity by modulating critical cellular processes in tumor cells [1, 22, 23]; however, the effects of reduced neddylation on immune functions in the context of tumor microenvironment are not well investigated yet. In this regard, different reports have described differential activities induced by neddylation inhibition in specific cancer conditions. Several observations described a decreased transcription of NF-κB-regulated genes and signaling pathways in TCR-stimulated T cells where NAE was inhibited, while others observed that CRLs may in fact negatively regulate TCR signalling and IL-2 synthesis [57, 58]. More recently, studies in chronic lymphocytic leukemia (CLL) indicated that patient-derived T cells treated with NAE inhibitors, showed significant differential expression of NF‐κB‐regulated genes and downregulated IL-2 expression/signalling during activation [59]. However, the impact of NAE inhibition on T-cell activation and proliferation was limited, suggesting that its targeting in the clinic is unlikely to be associated with impaired T-cell functionality. On the contrary, NAE inhibition redirected T-cell polarization suppressing induction of FoxP3^+^ Treg cells and stimulating anticancer activities [59]. Here, we add a novel piece of information describing how modulation of neddylation can regulate NK cell-activating ligands expression and sensitize MM cells to NK cell recognition and killing. The data we reported in this manuscript indicate that cell surface expression of the NKG2D ligands MICA and MICB is upregulated after inhibition of NAE in human MM cell lines, and in malignant plasma cells isolated from the bone marrow of MM patients (Figs. 1–3). The functional implication of this upregulation is an increased degranulation and NK cell-mediated killing of MM target cells in which NAE activity has been previously inhibited, with a mechanism dependent on the activation of the NKG2D receptor (Fig. 4). Concerning the molecular mechanisms involved, we observed a different regulation of MICA and MICB expression by modulation of NAE activity. After neddylation inhibition, MICA expression was upregulated at the transcriptional level. Using transient transfections, we identified a minimal MICA promoter fragment spanning −270bp from the translation start site still responsive to NAE inhibition (Fig. 5 A), and regulated by the TFs IRF4, IKZF1, and IKZF3, previously identified as IMiDs and Bromodomain and Extra-terminal Domain inhibitors (BETi) “druggable” transcriptional repressors of this gene in MM cells [35, 60]. We found IRF4 to be significantly downregulated at mRNA and protein levels after neddylation inhibition (Fig. 5B–F), as the result of NF-kB pathway inhibition (Fig. 6A–C), of which it represents a direct transcriptional target [43–45], configuring a role for neddylation in the regulation of the NF-kB/IRF4/MICA axis (Fig. 6D–F). Interestingly, as shown in Suppl. Figure 5, MLN4924 also induced a significant repression of IKZF3, with no relevant effects on IKZF1 expression, indicating a different regulation of these genes by neddylation in MM. Neddylation inhibition regulated in a different way MICB cell surface expression, with the absence of relevant changes of its mRNA levels (Fig. 2 and Fig. 3C). Indeed, as shown in Fig. 7A and B, we found a significant redistribution of MICB from the inner to the cell surface in SKO-007(J3) cells treated with MLN4924, and this was not related to increased levels of the ligand in the whole cell (Fig. 7C), suggesting the involvement of post-translational mechanisms in the upregulation of MICB expression on the cell surface. In this regard, NKG2DL expression can be regulated by different post-translational mechanisms [48]. In particular, active intracellular trafficking and recycling can regulate the expression of MICB [49]. Critical functions of CRLs in the endocytic system have been described; an example is Cullin3 that can be specifically activated by neddylation at the plasma membrane where it has been found to be associated with vesicular markers for intracellular trafficking [61]. Moreover, Cullin3 has been described as a regulator of the endolysosomal pathway [62] and more recently, a Speckle Type BTB-POZ Protein Like (SPOPL)/Cullin-3 ubiquitin ligase complex has been shown to regulate endocytic trafficking by targeting EPS15 at endosomes [63]. Interestingly, a dominant-negative mutant of EPS15 (EH29) has been shown to increase the proportion of MICB at the plasma membrane, inhibiting its active internalization [64]. Further investigation will be needed to characterize the involvement of specific CLR-mediated activities underlying MICB intracellular distribution. While complete ablation of neddylation appears to be clinically efficacious [65], in the last few years novel small-molecule inhibitors have been developed and investigated in order to reduce possible toxic effects mediated by complete ablation of CRLs activity. In an effort to maintain efficacy and reduce toxicity, targeting protein–protein interactions of the neddylation complexes has been pursued as a potential strategy to selectively inhibit the activity of individual CRLs. Recently, crystal structures of DCN1 (a subunit of a multiprotein E3 ligase for the ubiquitin-like protein NEDD8) and its binding partners UBE2M/UBC12 (an E2 neddylation enzyme) suggested that it may be suitable for the design of tailored small-molecule inhibitors [52, 53]. In this context, we investigated the activity of the pharmacologic inhibitor of the DCN1-UBE2M complex, NAcM-OPT, described to selectively reduce the steady-state neddylation of Cullin-1 and Cullin-3 with respect to other Cullins [52, 53]. Treatment of SKO-007(J3) cells with NAcM-OPT significantly increased cell surface expression of MICA and MICB, as detected by flow-cytometry (Suppl. Figure 6A, B and F, G); in this experimental setting, we also observed a significant repressive activity on IRF4 and IKZF3 expression (Suppl. Figure 6D, E), although of a lower level than that observed using MLN4924. Interestingly, these data are in agreement with the lower activity of NAcM-OPT compared to MLN4924 in different cancer cell lines using this DCN1 inhibitor [53, 66], suggesting a need to further improve the binding affinities to DCN1 and the efficacy for these novel class of compounds. Further experiments will be needed to extend and confirm these observations in primary isolated MM cells from patients. Finally, an interesting interplay between neddylation inhibition, IMiDs and expression of NKG2DL is described in this work, concerning the possibility to improve the activity of immunomodulatory drugs in MM therapy. The previous observations that 1) combination of Lenalidomide and Bortezomib was routinely used in the clinic with reported synergy [55, 67, 68], 2) CRBN can be targeted and degraded by SCF^Fbxo7^ E3-ubiquitin ligase [54] and 3) proteasome inhibitors (Bortezomib) as well as neddylation inhibitors (MLN4924) can decrease degradation of CRBN, enhancing sensitivity to IMiDs [55], led us to translate these findings in terms of increased efficacy of MLN4924 + Lenalidomide combination on promoting NKG2DL expression. As shown in Fig. 8A and B, we confirmed in our model that MLN4924 significantly increases CRBN protein expression; in addition, MLN4924 downregulated both mRNA and protein levels of the transcription factor MEIS2 (Fig. 8C and D), previously identified as an endogenous cellular substrate of the E3-ubiquitin ligase complex CRL4-cereblon (CRL4^CRBN^) [56], and recently described by our group as a regulator of IMiDs activity in MM, given its ability to regulate the expression of IRF4 and IKZF3 [47]. We found that pretreatment of SKO-007(J3) cells with MLN4924 for 24 h followed by stimulation with Lenalidomide, further increased MICA mRNA and cell surface expression, and further downregulated cellular levels of the MICA repressor IRF4, confirming the hypothesis (Fig. 8E-H). These data can further extend the preclinical and clinical observations of enhanced sensitivity to IMiDs after treatment with neddylation or proteasome inhibitors in MM, adding novel information on how their cooperation increases NKG2DL expression. In the clinical practice, Bortezomib is administered to MM patients once or twice a week, while Lenalidomide is taken daily [68], so part of the efficacy of this combination therapy could possibly rely on the mechanisms described above and, in this scenario, NAE inhibitors could represent an interesting alternative to proteasome inhibitors to be coupled with Lenalidomide. Further investigation will be needed to confirm these observations in primary MM cell from patients and to verify the functional therapeutic efficacy of the combo with IMiDs.

In summary, inhibition of neddylation sensitizes MM to NK cell recognition/killing, providing novel insights on the immuno-mediated antitumor activities of neddylation inhibition in MM.

## Materials and methods

### Cell lines and clinical samples

Human myeloma cell lines SKO-007(J3), RPMI-8226, U266, ARP-1, and JJN-3 have been already described [36, 47] and were kindly provided by Prof. P. Trivedi (University of Rome, Sapienza, Italy) and by Prof. N. Giuliani (University of Parma, Italy). After thawing, cells were cultured at 37 °C and 5% CO_2_ in RPMI 1640 supplemented with 15% FCS, 2 mM L-glutamine, 100 U/ml penicillin and 100 U/ml streptomycin (complete medium) for no longer than 4 weeks and tested for mycoplasma monthly (EZ-PCR Mycoplasma Test Kit, Biological Industries - Kibbutz Beit Haemek, Israel). These cell lines were authenticated by IRCCS Azienda Ospedaliera Universitaria San Martino-IST, S.S. Banca Biologica e Cell factory (Genova, IT). The human 293 T embryonic kidney cells were purchased from ATCC and were maintained in Dulbecco’s modified Eagle’s supplemented with 10% FCS, 2 mM L-glutamine, 100 U/ml penicillin, and 100 U/ml streptomycin. Bone marrow samples from MM patients were managed at the Division of Hematology, Department of Cellular Biotechnologies and Hematology, University of Rome, Sapienza, Italy (Table 1). Informed consent in accordance with the Declaration of Helsinki was obtained from all patients, and approval was obtained from the Ethics Committee of the Sapienza University of Rome (Rif. 3373). Bone marrow aspirates were processed as already described in [35]. In some experiments, myeloma cells were enriched using anti-CD138 magnetic beads (Miltenyi Biotec. S.R.L. Bologna, IT) and more than 90% of the purified cells expressed CD138 and CD38. Primary cultured human NK cells were obtained after 10-day co-cultures of PBMCs with irradiated Epstein-Barr virus-positive (EBV^+ ^) RPMI 8866 lymphoblastoid cell line as described in [69]. On day 10, the cell population was routinely >90% CD56^+ ^CD16^+ ^CD3^−^, as assessed by immunofluorescence and flow-cytometry analysis.

### Reagents and Antibodies

The NAE inhibitor MLN4924 and the DCN1 inhibitor NAcM-OPT were purchased from Selleckchem.Com (Houston, Texas, USA). Lenalidomide was purchased from BioVision Inc. (Milpitas, California USA). The final concentration of DMSO in all experiments was <0.1%. The following monoclonal antibodies (mAbs) were used for immunostaining or as blocking Abs: anti-MICA (MAB1300), anti-MICB (MAB1599), anti-ULBP-1 (MAB1380), anti-ULBP-2/5/6 (MAB1298), anti-ULBP-3 (MAB1517), and anti-NKG2D (MAB139) were purchased from R&D System (Minneapolis, MN, USA). Anti-MHC class I (W6/32) was purchased from American Type Culture Collection (ATCC, Manassas, VA, USA). Goat antimouse (GAM)-APC IgG [(Allophycocyanin-conjugated AffiniPure F(ab’)2 Fragment Goat AntiMouse IgG (H + L)] was purchased from Jackson ImmunoResearch Laboratories Inc. (West Grove, Pennsylvania, USA). Anti-CD138-FITC (M15) and anti-CD38-APC (HIT2) were purchased from BD Biosciences (Franklin Lakes, New Jersey, USA). PE/APC GAM IgG (Poly4053) was purchased from BioLegend (San Diego, California, USA). Anti-MICA-PE (FAB1300P), anti-MICB-PE (FAB1599P), Anti-MICA-APC (FAB1300A) and anti-MICB-APC (FAB1599A) were purchased from R&D System (Minneapolis, MN, USA). Anti-CD107a/PE and APC (H4A3), CD56/PE and APC (NCAM16), CD16/PerCP-Cy5.5 (3G8) and anti-CD3/FITC (SK7), were purchased from BD Biosciences. Antimouse IgG1-FITC, -PE, or -APC (MOPC-21) were purchased from BioLegend (San Diego, California, USA).

### Flow cytometry

SKO-007(J3), RPMI-8226, U266, ARP-1, and JJN-3 cells were cultured in 6-well tissue culture plates at 2 × 10^5^ cells/ml. The expression of the different NK cell-activating ligands was analyzed by immunofluorescence staining using unconjugated mAbs followed by secondary GAM-APC [(Allophycocyanin-conjugated AffiniPure F(ab’)2 Fragment Goat AntiMouse IgG (H + L) (Jackson ImmunoResearch Laboratories Inc., Baltimore Pike West Grove, PA, United States)]. In all experiments, cells were stained with Propidium Iodide (PI) (1 µg/ml) to assess cell viability (always higher than 90% after each treatment). Nonspecific fluorescence was assessed by using an isotype-matched irrelevant mAb (R&D System) followed by the same secondary antibody. To calculate the averages of the different mean fluorescence intensities (MFI) of each sample/treatment, the corresponding MFI of the control isotype (for each treatment) was subtracted from the MFI of the specific mAb/ligand (for the different treatments). Fluorescence was analyzed using a FACSCalibur flow cytometer (BD Biosciences) and data were analyzed using FlowJo Flow Cytometric Data Analysis Software (FlowJo—Ashland, OR, USA).

Plasma cells (PCs) from bone marrow aspirates were cultured in RPMI 1640 complete medium supplemented with the addiction of IL-3 (20 ng/ml) and IL-6 (2 ng/ml) at 1 × 10^6^ cells/ml. These cells were treated with MLN4924 or vehicle for 48 h. Cells were stained with APC/PE-conjugated mAbs. The analysis of NK cell-activating ligands has been done by gating on the CD38^+ ^CD138^+ ^PCs. In this case, fluorescence was analyzed using a FACS Canto II flow cytometer (BD Biosciences). Degranulation assays were performed using primary cultured human NK cells (CD3^-^CD56^+ ^CD16^+ ^) (cell purity was >80%). For CD107a cell surface expression, NK cells were co-cultured with untreated or MLN4924-treated SKO-007(J3) cells (Effector:Target E:T ratio of 2.5:1) for 3 h at 37 °C in the presence of PE-conjugated anti-CD107a mAb; 50 µM Monensin (Golgi-stop; Sigma-Aldrich) was added after the first hour. In these experiments, cells were washed after treatment with the drug in complete medium before co-culture. At the end of stimulation, conjugates were stained with APC-conjugated anti-CD56 mAb (BD Biosciences) and analyzed using a FACS Canto II flow cytometer (BD Biosciences). In some experiments, NK cells were pretreated for 20 min at room temperature with anti-NKG2D neutralizing mAb. When patient-derived plasma cells were used as targets, autologous CD138^−^ bone marrow cells were cultured for 2 days in complete medium, supplemented with 200 U/ml IL-2, and used as source of effector cells. Drug-treated and untreated patient-derived plasma cells were washed twice in complete medium and incubated with CD138^−^ bone marrow cells at Effector:Target (E:T) ratio of 2.5:1, in a U-bottom 96-well tissue culture plate in complete medium at 37 °C and 5% CO2 for 2 h. Thereafter, cells were washed with PBS and incubated with anti-CD107a/APC for 45 min at 4 °C. Cells were then stained with anti-CD3/FITC, anti-CD56/PE, and anti-CD16/PerCP-Cy5.5 to gate the CD3^-^CD56 ^+ ^CD16 ^+ ^NK cell population. Fluorescence was analyzed using a FACS Canto II flow cytometer (BD Biosciences) and data were analyzed using FlowJo Flow Cytometric Data Analysis Software (Tree Star). To evaluate NK cell-mediated cytotoxicity, SKO cells (untreated or treated with MLN4924) were incubated with cultivated NK cell from healthy donors, previously labeled with 5(6)-Carboxyfluorescein diacetate N-succinimidyl ester (CFSE) (2,5 µM) (Sigma-Aldrich, St Louis, MO), at different Effector:Target (E:T) ratio, for 4 h at 37 °C. Cells were then washed with PBS 1% BSA and stained with 7-Aminoactinomycin D (7-AAD) (Sigma-Aldrich, St Louis, MO) at the final concentration of 5 µg/ml for 20 min at 4 °C. Specific lysis of target cells has been analyzed using a FACS Canto II flow cytometer (BD Biosciences). Flow cytometric analysis was performed using FlowJo Flow Cytometric Analysis Software (FlowJo—Ashland, OR, USA). To evaluate the cell cycle, MM cell lines were harvested after 48 h of MLN4924 treatment, washed in PBS with 0.1% sodium azide, fixed in cold 70% ethanol, and incubated at −20 °C o/n. Thereafter, to remove ethanol and precipitated protein, cells were washed twice with PBS and then incubated with a solution containing propidium iodide (50 μg/mL) and RNAse (100 μg/mL) for 30 min at R/T. Cells were acquired using a FACSCalibur flow cytometer (BD Biosciences). Flow cytometric analysis was performed using FlowJo Flow Cytometric Analysis Software (FlowJo—Ashland, OR, USA).

### Apoptosis analysis

Apoptotic cell death was evaluated using APC Annexin-V Apoptosis Detection Kit (BioLegend). Briefly, MM cell lines or purified CD138^+ ^cells from patients, were cultured in six-well tissue culture plates at 2 × 10^5^ cells/ml (or 1 × 10^6^ cells/ml for patient’s MM cells), untreated or treated with MLN4924 for 72 h as described above. Cells were then stained using Annexin-V/APC according to the manufacturer’s instruction. Flow cytometric analysis was performed using a FACS Canto II flow cytometer (BD Biosciences) and data were analyzed using FlowJo Flow Cytometric Data Analysis Software (FlowJo—Ashland, OR, USA).

### Plasmids

The retroviral vector for IkBα-DN (S [32A]/S [36A]) in pMSCV-Neo (Murine Stem Cell Virus-Neomycin) was provided by J. Hischott (Pasteur Institute-Italy, Istituto Pasteur-Fondazione Cenci Bolognetti—Rome, Italy). The expression vector coding for the dominant-negative mutant of the repressor IKBα (S32A and S36A) in pRc-CMV was provided by A. Israel (Institut Pasteur, Paris, France). −1.2 kB MICA/Luc and −270bp MICA/Luc have been already described in [35].

### DNA transfections, virus production, and in vitro transduction

Transient transfections of SKO-007(J3) cells (5 × 10^6^) were carried out with 6 µg of the indicated 5’-promoter fragment (as described above) using Bio-Rad (Hercules, California, United States) Gene Pulser® II (0.4 cm cuvettes, 210 V, and 950 µF) in RPMI 1640. To reduce variability due to different transfection efficiency, cells were transfected in single batches and then separated into different drug treatment groups. A pTK-Renilla expression vector (1 µg) was cotransfected to normalize DNA uptake. After 2 h, cells were treated with MLN4924 or vehicle for 48 h. Cells were harvested, and protein extracts were prepared for the Luciferase and Renilla assays. The protein concentration of lysates was quantified through the BCA method (Pierce, Rockford, IL, USA). Luciferase and Renilla activities were quantified using the Dual-Luciferase Reporter Assay and the Glomax Multi Detection System (Promega) following the manufacturer’s instructions. When cells were cotransfected with pRc-CMV IkBα-DN (S [32A]/S [36A]), 6 µg of Luc-vector and 2 µg of expression vector were used, together with the pTK-Renilla expression vector. For retrovirus production, viral vectors were cotransfected together with the packaging vectors pVSVG and pGAG-Pol-Env into 293 T cells using Lipofectamine 2000 (Life Technologies). After transfection, cells were placed in a fresh medium. After a further 48-h culture, virus-containing supernatants were harvested, filtered, and used immediately for infections. Infections were performed on 1.5 × 10^6^ SKO-007(J3) cells in 2 ml virus-containing supernatants with Polybrene (8 μg/ml) (Hexadimethrine bromide—Sigma-Aldrich) for 2 h as previously described [69]. For IkBα-DN (S [32A]/S [36A]) stable clones, after infection cells were allowed to expand for 24 h and then selected for neomycin (1 mg/ml) resistance [70].

### mRNA detection and quantitative real-time polymerase chain reaction (qRT-PCR)

Total RNA was extracted using TRI Reagent™ Solution (Invitrogen, Thermo Fisher Scientific, Waltham, Massachusetts, USA) or Total RNA Mini kit (Geneaid Biotech, New Taipei City, Taiwan), according to manufacturer’s instructions. The concentration and quality of the extracted total RNA were determined by measuring light absorbance at 260 nm (A260) and the ratio of A260/A280. Reverse transcription was carried out in a 25 µl reaction volume with 2 µg of total RNA according to the manufacturer’s protocol for M-MLV reverse transcriptase (Promega, Madison, Wisconsin, USA). cDNAs were amplified (TaqMan assays) in triplicate with primers for MICA (Hs00792195_m1), MICB (Hs00792952_m1), IRF4 (Hs01056533_m1), IKZF1 (Hs00958474_m1), IKZF3 (Hs00232635_m1), MEIS2 (Hs00542638_m1), CRBN (Hs00372271_m1), GAPDH (Hs02758991_g1) conjugated to the fluorochrome FAM (Applied Biosystems, Foster City, California, USA). The expression level was measured using the comparative Ct (threshold cycle) method. ΔCt was obtained by subtracting the Ct value of the gene of interest from the selected housekeeping gene (GAPDH) Ct value. In the present study, we used the ΔCt of the untreated sample as calibrator. The fold change was calculated according to the formula 2^-ΔΔCt^, where ΔΔCt was the difference between the ΔCt of the sample and the ΔCt of the calibrator (according to the formula, the value of the calibrator in each run is 1). The analysis was performed using the SDS version 2.4 software (Applied Biosystems). All PCR reactions were performed using an ABI Prism 7900 Sequence Detection system (Applied Biosystems).

### Confocal microscopy

SKO-007(J3) cells were plated on multichamber slides (Falcon) precoated with poly-L-lysine (Sigma-Aldrich) and let adhere by centrifugation at 100 g. Cells were then fixed with 4% paraformaldehyde and stained with 1 mg/ml of Alexa Fluor 594-conjugated Wheat Germ Agglutinin (W11262—Thermo Fisher Scientific) for 10 min at room temperature. Cells were treated with Glycine 0.1 M for 20 minutes to quench PFA and permeabilized with 0.1% Triton-X-100 for 5 min. Cells were stained with anti-MICB (MAB1599—R&D System) for 1 h followed by Alexa Fluor 488-conjugated goat antimouse IgG (A11001—Invitrogen, Life Technologies) for 1 h, all diluted in blocking buffer (PBS 0.01% Triton-X-100, 1% FBS). After extensive washing coverslips were mounted using SlowFade Gold Antifade Mountant with DAPI (S36938—Thermo Fisher Scientific). High-resolution images (800 × 800 pixel, 8 μs/pixel, zoom 2x) were acquired at room temperature using IX83 FV1200 MPE laser-scanning confocal microscope with a 60×/1.35 NA UPlanSAPO oil immersion objective (all from Olympus, Shinjuku, Tokyo, Japan) as previously described [71, 72]. Images were processed with Fiji ImageJ software. Data quantification and colocalization analysis were performed with Fiji ImageJ software.

### Western-blot analysis

For Western-Blot analysis, SKO-007(J3) cells or patient-derived myeloma cells (isolated using anti-CD138 magnetic beads Miltenyi Biotec) were pelleted, washed once with cold phosphate-buffered saline, resuspended in lysis buffer [1 mM EDTA, 50 mM Tris-HCl pH 7.6, 150 mM NaCl, 0,2% Triton-X-100, 0,3% Nonidet P-40 (NP-40), 50 mM NaF, 1 mM Na_3_VO_4_, 1 mM PMSF, Protease Inhibitor Cocktail 1X (Sigma-Aldrich, St. Louis, Missouri, USA), Phosphatase Inhibitor Cocktail 3 1X (Sigma-Aldrich)] and then incubated 30 min on ice. The lysates were centrifuged at 16,000 g for 20 min at 4 °C and the supernatants were collected as whole-cell extract. Protein concentration was determined through the BCA method (Pierce—Thermo Fisher Scientific, Milan, IT). Nuclear proteins were prepared as described in [73]. The protein concentration of nuclear extracts was determined as described above. Fifteen to 30 μg of cell (or nuclear) extracts were run on 8 or 10% denaturing SDS-polyacrylamide gels. Proteins were then electroblotted onto Amersham™ Protran™ nitrocellulose membranes (GE Healthcare Life Science, Chicago, Illinois, USA), stained with Ponceau to verify that similar amounts of proteins had been loaded in each lane, and blocked with 5% BSA in TBST buffer. Immunoreactive bands were visualized on the nitrocellulose membranes, using horseradish-peroxidase-linked/coupled donkey antirabbit (NA934V) or sheep antimouse (NA931V) IgG (Amersham, GE Healthcare Life Science) and the ECL substrate WESTAR ηC ULTRA 2.0 (Cyanagen, Bologna, Italy), following the manufacturer’s instructions. Antibodies against IRF4 (H-140), IKZF1 (H-100), IKZF3 (O-21), pIκBα (B9), p65/RelA (C-20), p21 (F-5), and MEIS2 (63-T) were purchased from Santa Cruz Biotechnology (Dallas, Texas, USA). Antibody against CRBN (HPA045910) and β-Actin (AC-15) were purchased from Sigma-Aldrich. The ImageLab software version 5.2.1 was used for image acquisition and densitometric analysis of the gels using a ChemiDoc™ MP System (Bio-Rad, Hercules, California, United States). Target protein levels were referred to β-Actin, chosen to normalize protein expression.

### Statistical analysis

Error bars represent SEM. Data have been evaluated by paired Student’s *t* test using GraphPad Prism 8 and a *P* < 0.05 was considered statistically significant.

## Supplementary information

Suppl. Figure Legends

Suppl. Fig. 1

Suppl. Fig. 2

Suppl. Fig. 3

Suppl. Fig. 4

Suppl. Fig. 5

Suppl. Fig. 6

## Data Availability

All data generated or analyzed during this study are included in this published article and its supplementary information files.
