# Supplementary material for: Immunomodulatory effect of NEDD8-activating enzyme inhibition in Multiple Myeloma: upregulation of NKG2D ligands and sensitization to Natural Killer cell recognition
Source: Cell Death Dis. 2021 Sep 4;12(9):836. doi: 10.1038/s41419-021-04104-w (PMC8418610; doi:10.1038/s41419-021-04104-w)
Supplement: Supplementary file 1 — Suppl. Figure Legends [file 41419_2021_4104_MOESM1_ESM.docx]

**Immunomodulatory effect of NEDD8-activating enzyme inhibition in Multiple Myeloma: upregulation of NKG2D ligands and sensitization to Natural Killer cell recognition.**

**Supplementary Figures Legends**

Suppl. Fig. 1 – **Effect of** **MLN4924 on MICA and MICB expression in ARP-1 and JJN-3 MM cell lines.** MICA, and MICB cell surface expression was analyzed by flow cytometry on ARP-1 and JJN-3 cells treated with MLN4924 (50 and 100 nM) for 72h. In this experimental setting, a lower range of concentrations (50 to 100 nM) of MLN-4924 was used due to the higher sensitivity of these cells to this drug. Representative overlays are shown. Histograms represent the average of the mean fluorescence intensity (MFI) values of the indicated ligand (with treatment-specific isotype control MFI subtracted out). The MFI of MICA and MICB were calculated based on at least three independent experiments and statistical significance (± SEM) was evaluated by paired Student *t* test (**P*< 0.05).

Suppl. Fig. 2 - **Effect of MLN4924 treatment on ULBPs expression in MM cell lines.** ULBPs cell surface expression was analyzed by flow cytometry on SKO-007(J3), RPMI-8226 and U266 cells treated with MLN4924 200 nM for 72h. Histograms represent the average of the mean fluorescence intensity (MFI) values of the indicated ligand (with treatment-specific isotype control MFI subtracted out). The MFI of the different ULBPs were calculated based on at least three independent experiments and statistical significance (± SEM) was evaluated by paired Student *t* test (**P*< 0.05).

Suppl. Fig. 3 - **Annexin-V assay on MM cell lines treated with MLN4924**. SKO-007(J3), RPMI8226 and U266 cells untreated or treated with MLN4924 200 nM for 72h. A) Representative and B) the average of % Annexin-V positive cells (three independent experiments) is shown.

Suppl. Fig. 4 - **Annexin-V staining on purified MLN4924-treated CD138^+^ cells from MM patients**. Purified cells were stimulated with MLN4924 as indicated in Figure 3 and then stained with Annexin-V/APC. The % Annexin-V positive cells is indicated in the histograms.

Suppl. Fig. 5 - **Expression of IKZF3 but not IKZF1 is inhibited by MLN4924 in MM cells.**

A, C) Western blot analysis of IKZF3 and IKZF1 in SKO-007(J3) cells untreated or treated with MLN4924 for 48h. β-Actin was used as protein loading control. Data are representative of one out of three independent experiments. Densitometric analysis of the reported Western blot is shown. B, D) Real Time PCR analysis of total mRNA obtained from SKO-007(J3) cells, untreated or treated with the indicated concentration of MLN4924 for 48h. Data, expressed as fold change units, were normalized to GAPDH, and referred to the untreated sample considered as calibrator, represent the mean (± SEM) of 4 experiments (**P*< 0.05). E) Real Time PCR analysis of total mRNA obtained from purified CD138^+^ cells untreated or treated with MLN4924 (400 nM) as described in Figure 3 for 48h in complete medium supplemented with 20 ng/ml IL-3 and 2 ng/ml IL-6. Data, expressed as fold change units, were normalized to GAPDH, and referred to the untreated cells considered as calibrator. F, G) Western blot analysis of IKZF3 in MM cells purified from two patients (CD138^+^ cells) untreated or treated with MLN4924 (200 and 400 nM) for 48h. β-Actin was used as protein loading control. Densitometric analysis of the reported Western blot is shown.

Suppl. Fig. 6 - **DCN1 inhibition upregulates MICA/B expression in SKO-007(J3) cells.**

A) MICA cell surface expression was analyzed by flow cytometry on SKO-007(J3) cells treated with NAcM-OPT (10 µM) for 72h. A representative overlay is shown. B) Histograms represent the average of the mean fluorescence intensity (MFI) values of the indicated ligand (with treatment-specific isotype control MFI subtracted out). The MFI of MICA was calculated based on at least four independent experiments and statistical significance was evaluated by paired Student *t* test (**P*< 0.05). C) Real Time PCR analysis of total mRNA obtained from SKO-007(J3) cells, untreated or treated with the indicated concentrations of NAcM-OPT as described above, for 48h. Data, expressed as MICA fold change units, were normalized to GAPDH, and referred to the untreated sample considered as calibrator, represent the mean (± SEM) of 3 experiments (**P*< 0.05). D, E) Real Time PCR analysis of total mRNA obtained from SKO-007(J3) cells, untreated or treated with the indicated concentrations of NAcM-OPT as described above, for 48h. Data, expressed as fold change units, were normalized to GAPDH, and referred to the untreated cells considered as calibrator, represent the mean (± SEM) of 3 experiments (**P*< 0.05). F) MICB cell surface expression was analyzed by flow cytometry on SKO-007(J3) cells treated with NAcM-OPT (10 µM) for 72h. A representative overlay ais shown. G) Histograms represent the average of the mean fluorescence intensity (MFI) values of the indicated ligand (with treatment-specific isotype control MFI subtracted out). The MFI of MICB was calculated based on at least four independent experiments and statistical significance was evaluated by paired Student *t* test (**P*< 0.05). H) Real Time PCR analysis of total mRNA obtained from SKO-007(J3) cells, untreated or treated with the indicated concentrations of NAcM-OPT as described above, for 48h. Data, expressed as MICB fold change units, were normalized to GAPDH, and referred to the untreated cells considered as calibrator, represent the mean (± SEM) of 3 experiments (**P*< 0.05).
