# Supplementary figures and images for: Immunomodulatory effect of NEDD8-activating enzyme inhibition in Multiple Myeloma: upregulation of NKG2D ligands and sensitization to Natural Killer cell recognition
Source: Cell Death Dis. 2021 Sep 4;12(9):836. doi: 10.1038/s41419-021-04104-w (PMC8418610; doi:10.1038/s41419-021-04104-w)

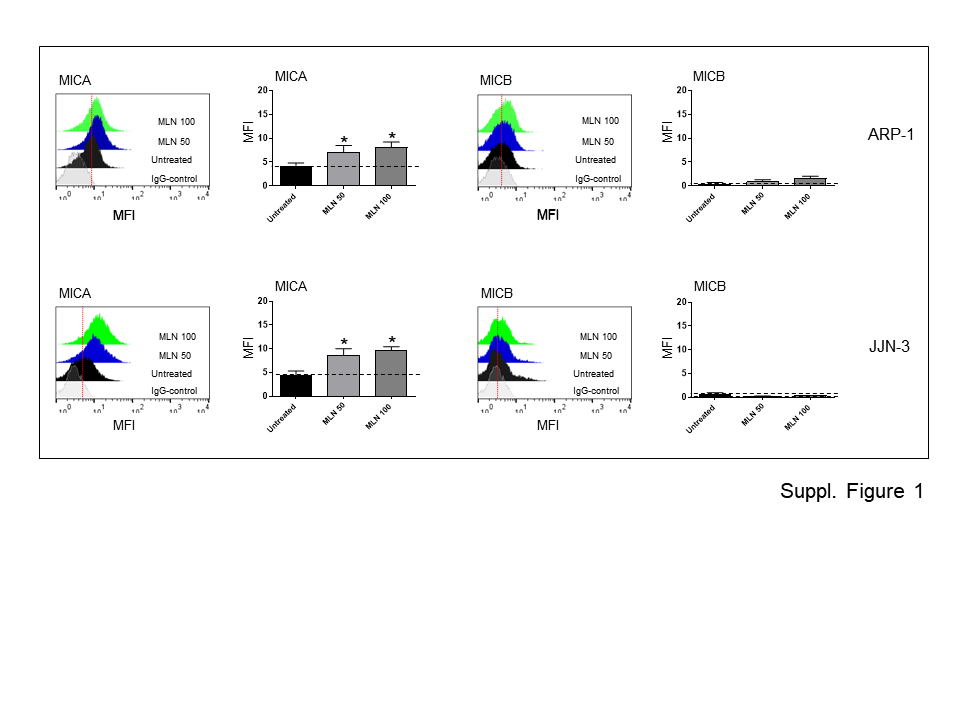

Supplement: Supplementary file 2 — Suppl. Fig. 1 [file 41419_2021_4104_MOESM2_ESM.png]

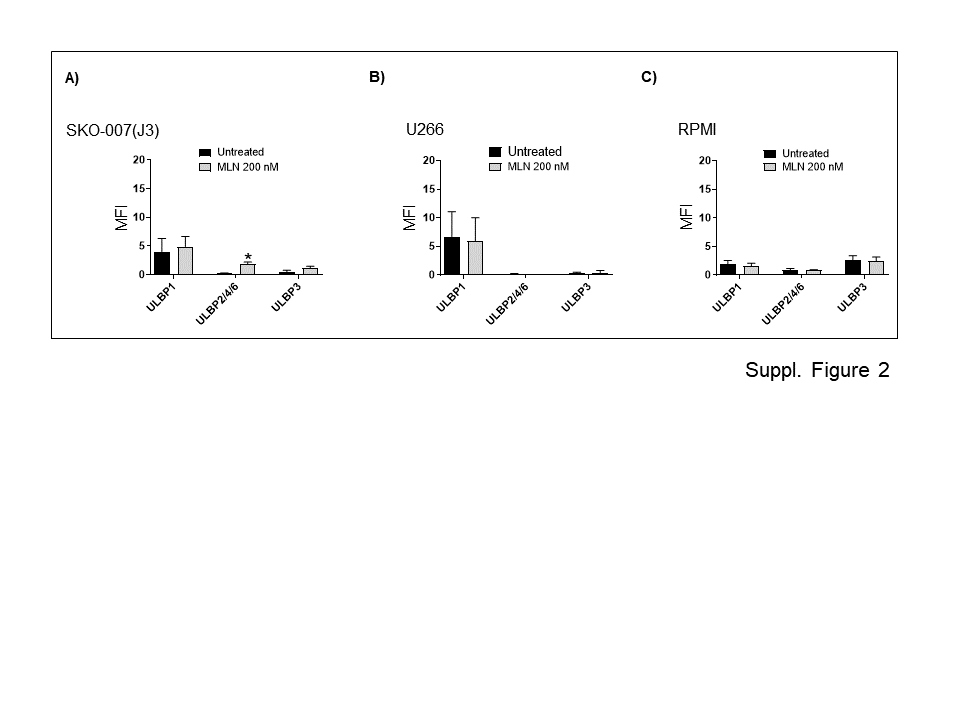

Supplement: Supplementary file 3 — Suppl. Fig. 2 [file 41419_2021_4104_MOESM3_ESM.png]

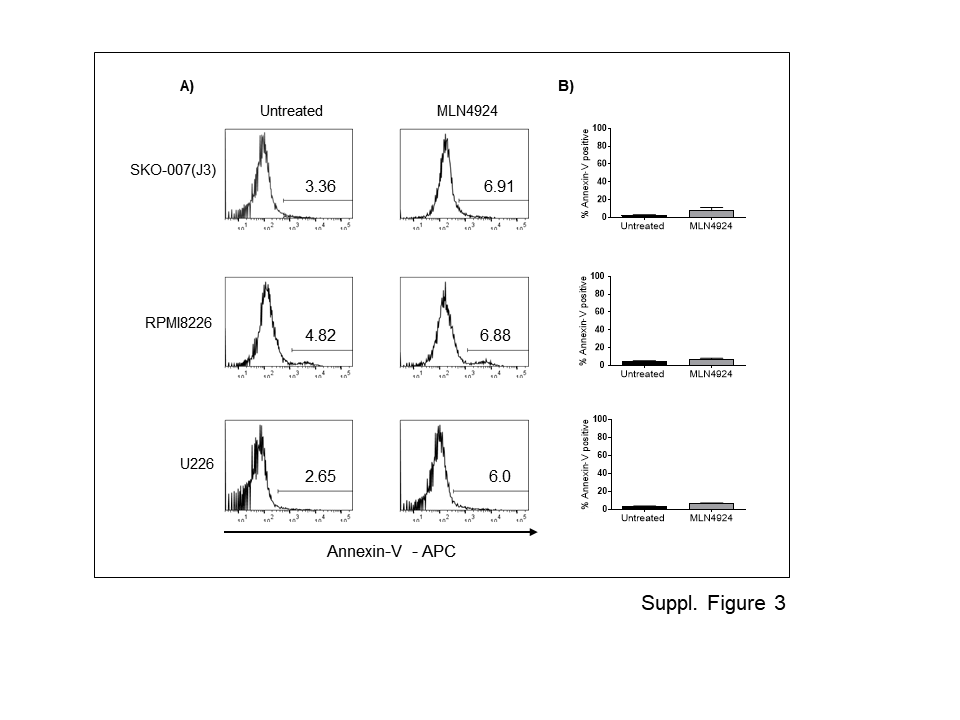

Supplement: Supplementary file 4 — Suppl. Fig. 3 [file 41419_2021_4104_MOESM4_ESM.png]

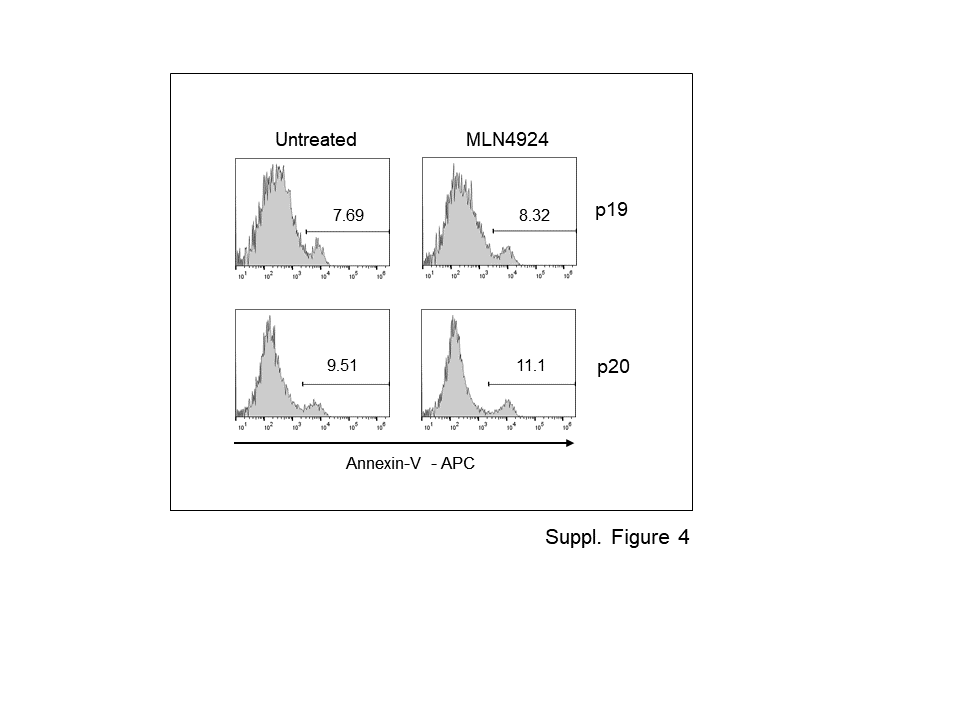

Supplement: Supplementary file 5 — Suppl. Fig. 4 [file 41419_2021_4104_MOESM5_ESM.png]

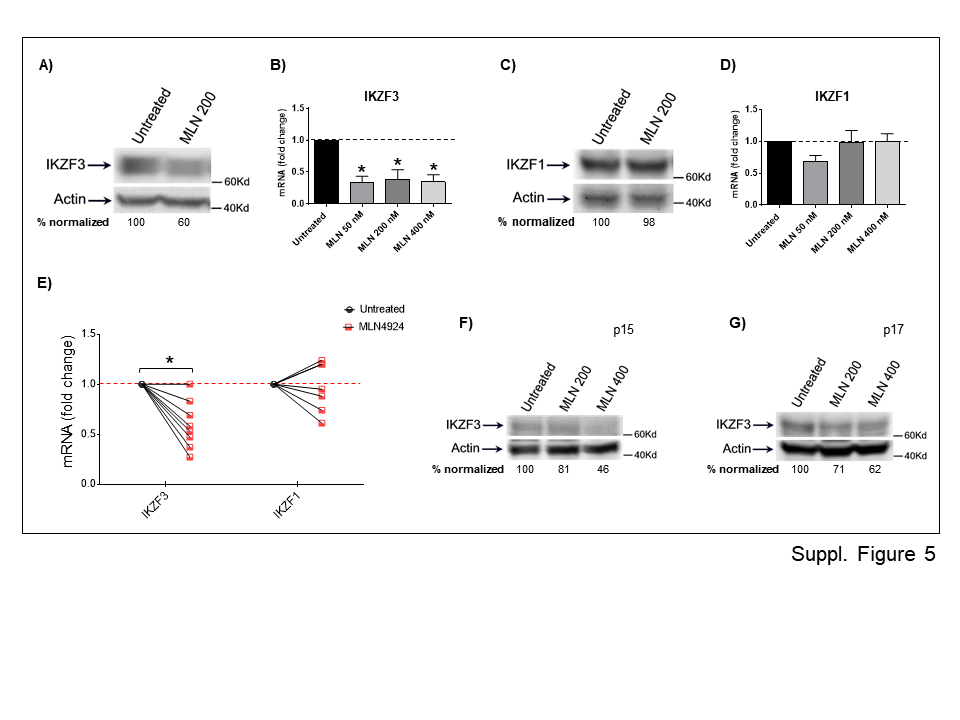

Supplement: Supplementary file 6 — Suppl. Fig. 5 [file 41419_2021_4104_MOESM6_ESM.png]

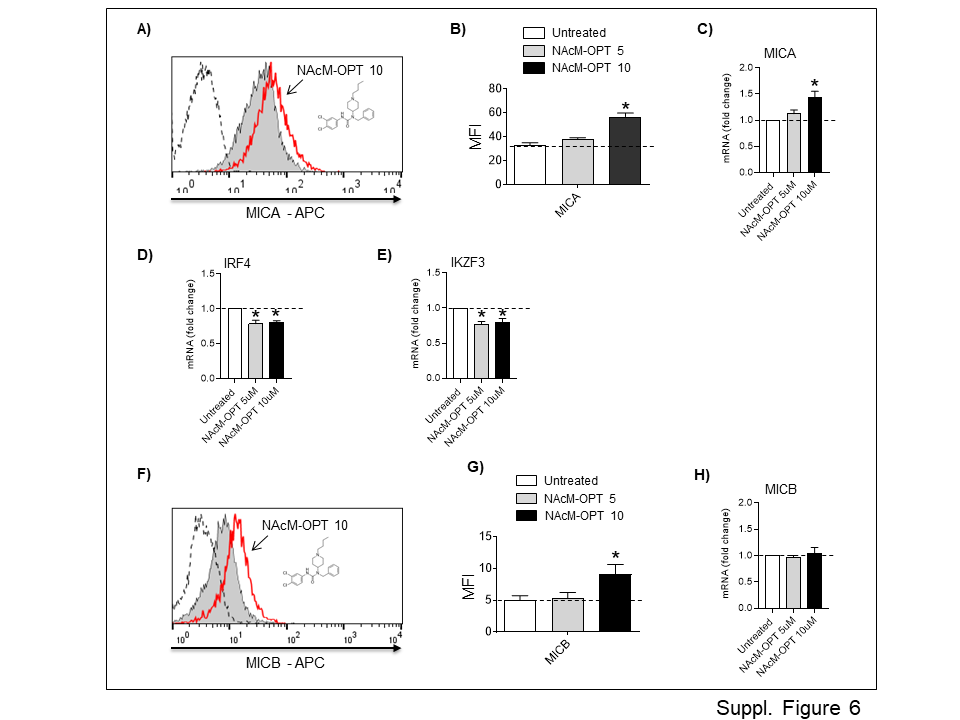

Supplement: Supplementary file 7 — Suppl. Fig. 6 [file 41419_2021_4104_MOESM7_ESM.png]
